# Comparison of cardiac function and structure after left atrial appendage occlusion without versus with ablation in patients with non-valvular atrial fibrillation: a retrospective study

**DOI:** 10.7150/ijms.95080

**Published:** 2024-07-01

**Authors:** Zhong-bao Ruan, Fei Wang, Ge-cai Chen, Jun-guo Zhu, Yin Ren, Li Zhu

**Affiliations:** Department of Cardiology, the affiliated Taizhou People's Hospital of Nanjing Medical University, Taizhou School of Clinical Medicine, Nanjing Medical University, Taizhou 225300, P.R. China.

**Keywords:** left atrial appendage occlusion, ablation, atrial fibrillation, cardiac structure, cardiac function.

## Abstract

The Aim of this study was to investigate the long-term impact of left atrial appendage occlusion (LAAO) on cardiac function and structure in patients with non-valvular atrial fibrillation (NVAF). 157 patients with NVAF who underwent LAAO or combined with ablation were included and divided into simple LAAO group or combined group. Long term impact of LAAO on cardiac function and structure were evaluated. Results showed that the procedures were performed successfully with 6.4% complications. During follow-up, there was a significant decrease of left atrial anteroposterior diameter (LAAD) at 6 months and a significant increase of left ventricular end-diastolic dimension (LVEDD) at 12 months after LAAO. A significant decrease in plasma N-terminal pro-brain natriuretic peptide (NT-proBNP) was noted at 3 months, 6 months and 12 months after procedure. There was a significant decrease of LAAD, LVEDD, left ventricular end-systolic dimension (LVESD) and NT-proBNP levels in combined group at 3 months, 6 months and 12 months post- procedure, while an increase of left ventricular ejection fraction (LVEF). Meanwhile, no significant change of LAAD, LVEDD, LVESD, NT-proBNP and LVEF was seen in simple LAAO group at 3 months follow-up, but a decrease of NT-proBNP during 6 months and 12 months follow-up. Compared with simple LAAO group, combined group was associated with a significant increase of residual flow. In conclusion, LAAO has no significant effect on cardiac structure and function but can significantly reduce NT-proBNP. The improvement of cardiac structure and function in combined therapy comes from the result of ablation, not LAAO.

## Introduction

Atrial fibrillation (AF) is one of the most common arrhythmias and the incidence increases with age 1. Thromboembolic complication is the leading cause of death and disability in patients with AF 2. Studies have shown that left atrial appendage (LAA) is the main cardioembolic site in non-valvular atrial fibrillation (NVAF) 3. Oral anticoagulation (OAC), warfarin or novel oral anticoagulants (NOACs), is currently one of the main methods for stroke prevention in patients with NVAF, but increasing the risk of bleeding. Left atrial appendage occlusion (LAAO) has now been shown to be a safe and effective alternative therapy to oral anticoagulation (OAC) for preventing stroke in NVAF patients with high risk 4, 5. Emerging evidence indicates that LAA is a contractile and compliant organ that involves in the regulation of pressures in the left atrium and plays an important role in the preservation of left ventricular filling. Meanwhile, it is one of the major hormone-producing sites, involves in neurohormonal secretion such as brain natriuretic peptide (BNP) and atrial natriuretic peptide (ANP) 6, 7, which can affect cardiac function and structure and prevent the development and progression of heart failure (HF) 8.

Regarding the physiological role of the LAA, whether occlusion of the LAA may have any deleterious effects on the human body is still unclear to medical science so far. There are several reports providing inconsistent results about the impact of LAAO on cardiac function and structure in NVAF patients 9-13. Therefore, in this study, we aimed to investigate the effect of LAAO on cardiac structure and function in patients with NVAF through long term follow-up after LAAO.

Radiofrequency catheter ablation (RFCA) or Cryoablation (Cryo), as the two mainstream methods for treating NVAF, can maintain sinus rhythm, improve survival rate, reduce readmission, and reverse cardiac dysfunction, especially for patients with concomitant heart failure. The combination strategy of LAAO for stroke prevention and catheter ablation for AF symptoms controlling was proposed and the feasibility and safety were proven 14, 15. However, the effect of combination therapy on cardiac function and structure has not been investigated. Therefore, the impact of combination therapy on cardiac function and structure was investigated at the same time. Meanwhile, comparison of cardiac function and structure after LAAO without versus with ablation in patients with NVAF was performed.

## Methods

### Study population

This study included 157 patients with NVAF who underwent Watchman LAAO or combination with radiofrequency catheter ablation (RFCA) / cryoballoon ablation (Cryo) at Jiangsu Taizhou People's Hospital from March 2018 to November 2020, and the diagnosis of NVAF was mainly based on the criteria listed in the 2020 ESC Guidelines for the diagnosis and management of atrial fibrillation developed in collaboration with the European Association for Cardio-Thoracic Surgery (EACTS) 16. All patients met the inclusion criteria: ① definite diagnosis of NVAF refractory to antiarrhythmic drugs; ② Age ≥ 18 years; ③ CHA2DS2-VASc score ≥ 2 (male) or ≥ 3 (female); ④ Intolerant or unwilling to long-term anticoagulant therapy or contraindicated with anticoagulant drugs; The exclusion criteria were as follows: ① Transesophageal echocardiography (TEE) or CT angiography (CTA) showed thrombus in left atrium or LAA before operation; ② left atrial anteroposterior diameter (LAAD) > 65 mm for LAAO or > 55 mm for radiofrequency ablation/cryoablation; ③ LAA ostium < 17 mm or > 31 mm; ④ patients with rheumatic or valvular heart disease; ⑤ history of stroke within 1 month; ⑥cardiac function class IV (NYHA class New York); ⑦ inadequate control of hypertension; ⑧ coagulopathy. A total of 157 subjects were included in the study after screening. The study was approved by the hospital ethics committee and all patients signed the informed consent form.

### Data Collection

The relevant laboratory tests (including routine blood tests, coagulation, biochemistry index, thyroid function and plasma N-terminal pro-brain natriuretic peptide (NT-proBNP) and routine electrocardiogram were performed preoperatively. Clinical data were collected including age, gender, body mass index (BMI) (Kg/m^2^), type of NVAF, New York Heart Association (NYHA) functional classification, medical history (including hypertension, diabetes mellitus, coronary artery disease, congestive heart failure/cardiac insufficiency, peripheral vascular disease, hepatic insufficiency, renal insufficiency), history of thromboembolism (including stroke/transient ischemic attack), history of hemorrhage, CHA2DS2-VASc score, HAS-BLED score, perioperative adverse events.

### TEE and CTA protocol

TEE (Philips EPIQ 7C) and CTA (Siemens 128-row SOMATOM Force, Germany) were performed to eliminate LA A thrombus and to calculate the orifice diameter and depth of LAA before the procedure. Meanwhile, the following parameters were collected by TEE including left atrial anteroposterior diameter (LAAD), left ventricular end-diastolic dimension (LVEDD), left ventricular end-systolic dimension (LVESD) and left ventricular ejection fraction (LVEF). The TEE operator was the same at baseline and follow up.

### LAAO procedure using the Watchman2.5 device

LAAO procedure was performed under deep intravenous anesthesia or general anesthesia and TEE monitoring. Following successful transseptal puncture, angiography of LAA was performed to measure the orifice diameter and depth of LAA. Based on the measured parameters on diameter and depth of LAA by TEE, CTA and digital subtraction angiography (DSA), oversizing by 4 mm-6 mm of the diameter of the LAA was selected as a suitable device size. The Watchman2.5 device was released when the conditions of a proper position with no or minimal (<5 mm) residual flow and a safety tug test were met by DSA and TEE.

### RFCA and Cryo procedure

In combined group, RFCA or Cryo was performed before LAAO. RFCA was used in 65 patients with persistent or paroxysmal atrial fibrillation. The left atrium and pulmonary vein models were constructed with CARTO three-dimensional mapping system (CARTO R 3; Biosense Webster, Irvine, CA, USA), pulmonary vein isolation (PVI) was performed in all patients with Coolflex catheters. For persistent atrial fibrillation, left atrial substrate mapping and homogenous ablation of low voltage areas were performed. Cryo was used in 21 patients with paroxysmal atrial fibrillation. A 28mm diameter of second generation cryoballoon (Arctie Front @ Cryoablation Catheter, Medtronic Inc., USA) was used for the purpose of PVI.

### Post-procedural anticoagulation

The therapy of anticoagulation combined with antiplatelet was recommended for 3 months following the procedure. Antiarrhythmic drugs were also used for 3 months in patients with combined procedure. Dual antiplatelet therapy with aspirin and clopidogrel was recommended for another 3 months if there were neither thromboembolism nor persistent peri-device leaks more than 5 mm detected by TEE follow-up. Then aspirin or clopidogrel was recommended indefinitely.

### Follow-up

Patients were followed up at 3 months, 6 months and 12 months post- procedure. During the follow-up, adverse events including thromboembolic events, bleeding events and mortality etc were recorded. TEE was performed and the plasma NT-proBNP was measured to evaluate the changes of cardiac function and structure. Residual leak and device-related thrombus (DRT) formation were also assessed by TEE.

### Statistical analysis

Statistical software SPSS 26.0 was used for analysis, Kolmogorov-Smirnov test was used to test whether the data were normal distribution. The normal distribution in the measurement data was presented as mean ± standard deviation, and paired sample t-test is used for comparison. Categorical data were presented as counts and percent and chi-square test were applied. A p value < 0.05 was considered statistically significant.

## Results

### Clinical baseline characteristics

**Figure 1:**
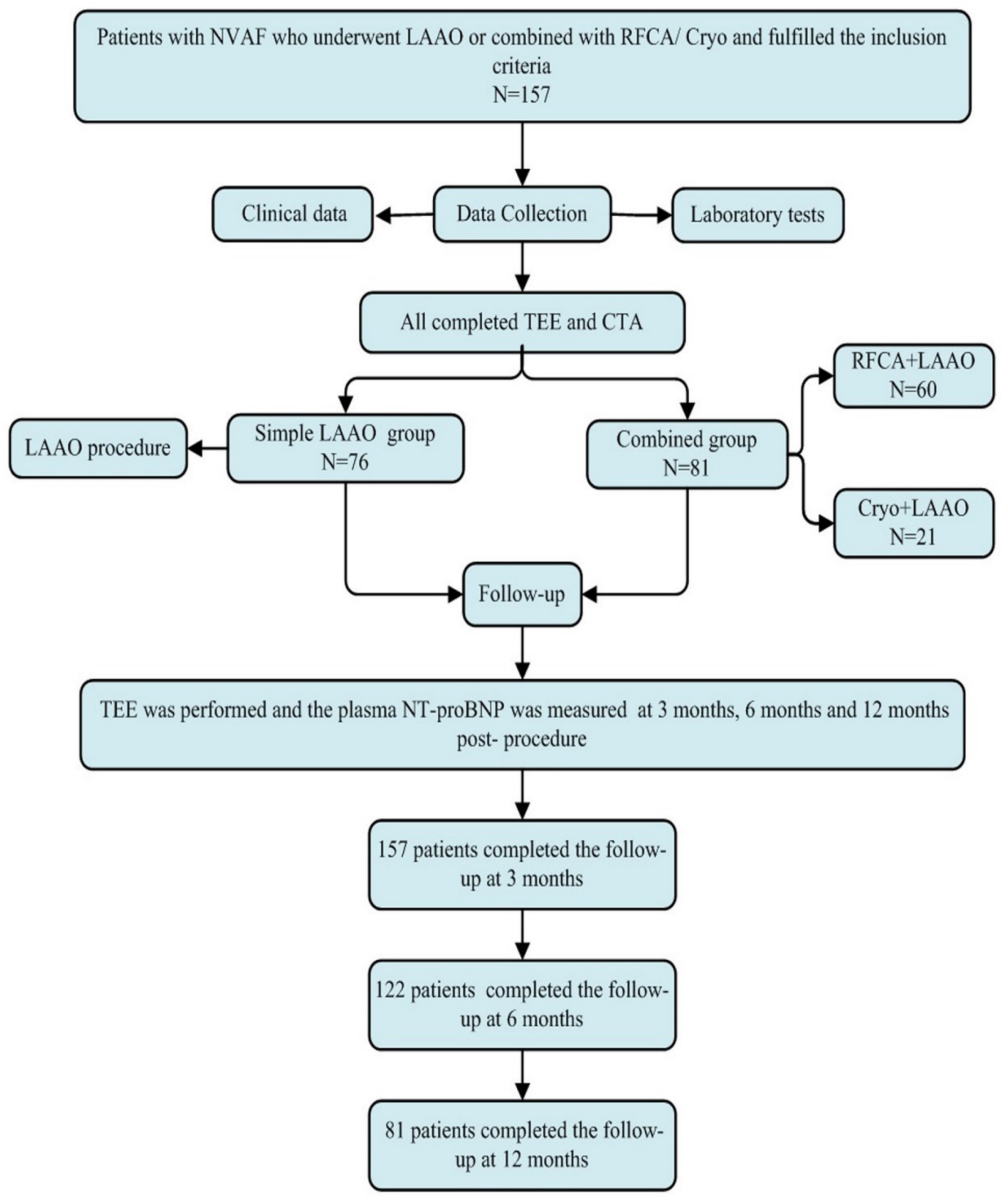
Flowchart of the study procedure. LAAO: left atrial appendage occlusion, Cryo: cryoballoon ablation, RFCA: radiofrequency catheter ablation, TEE: transesophageal echocardiography, NT-proBNP: n-terminal pro-brain natriuretic peptide.

Patient disposition was summarized in Figure 1. In this study, of the 157 patients, 76 underwent LAAO only (Simple LAAO group) and 81 underwent LAAO alongside ablation (Combined group). The mean age was 67.4 ± 8.9 years, the mean CHA_2_DS_2_-VASc score was 3.06 ± 1.06 and HAS-BLED score 2.78 ± 0.81. There were 109 (69.4%), 73 (46.5%), 38 (24.2%), 46 (29.3%), 35 (22.3%) and 5 (3.2%) of the patients suffering from hypertension, congestive heart failure, diabetes mellitus, prior stroke, coronary heart disease and bleeding history, respectively. The detailed clinical baseline characteristics were shown in Table 1.

### Assessment of the periprocedural parameters

The LAAO procedure with Watchman2.5 occluders or combined procedure was performed successfully in included patients. The LAA ostial diameter and depth measured by preoperative CTA (diameter: 23.6 ± 3.7 mm, depth: 25.1 ± 3.9 mm) was greater than those by TEE (diameter: 22.4 ± 3.6 mm, depth: 23.8 ± 3.6 mm) and DSA (diameter: 22.9 ± 3.7 mm, depth: 24.6 ± 3.7 mm). There were 11 (7.0%), 28 (17.8%), 49 (31.2%), 30 (19.1%) and 39 (24.8%) of all Watchman2.5 devices for 21 mm, 24 mm, 27 mm, 30 mm and 33 mm, respectively. There were 129 (82.2%) patients without no residual leak, 24 (15.3%) patients with residual leak less than 3 mm, 4 (2.5%) patients with residual leak between 3 mm and 5 mm. After LAAO, shoulder exposure happened in 31 cases (19.7%) and the mean compression ratio was (21.6 ± 4.6) %. There were 10 (6.4%) cases with complications, in which cardiac tamponade was found in 2 (1.3%) cases during and after the operation micropericardial effusion occurred in 5 (3.2%) cases. Detailed data are presented in Table 2.

### Impact of LAAO or combined with ablation on cardiac function and structure

As shown in Table 3, there were 157 patients, 122 patients and 81 patients completed the follow-up at 3 months, 6 months and 12 months, respectively. There was a significant decrease of LAAD at 6 months. Meanwhile, a significant increase of LVEDD was seen at 12 months after LAAO. No significant impact was indicated in LVESD and LVEF. However, the LAAO therapy was associated with a significant decrease in plasma NT-proBNP level at 3 months, 6 months and 12 months after operation.

### Comparison of the influence on cardiac function and structure between Simple LAAO group and Combined group

**Figure 2:**
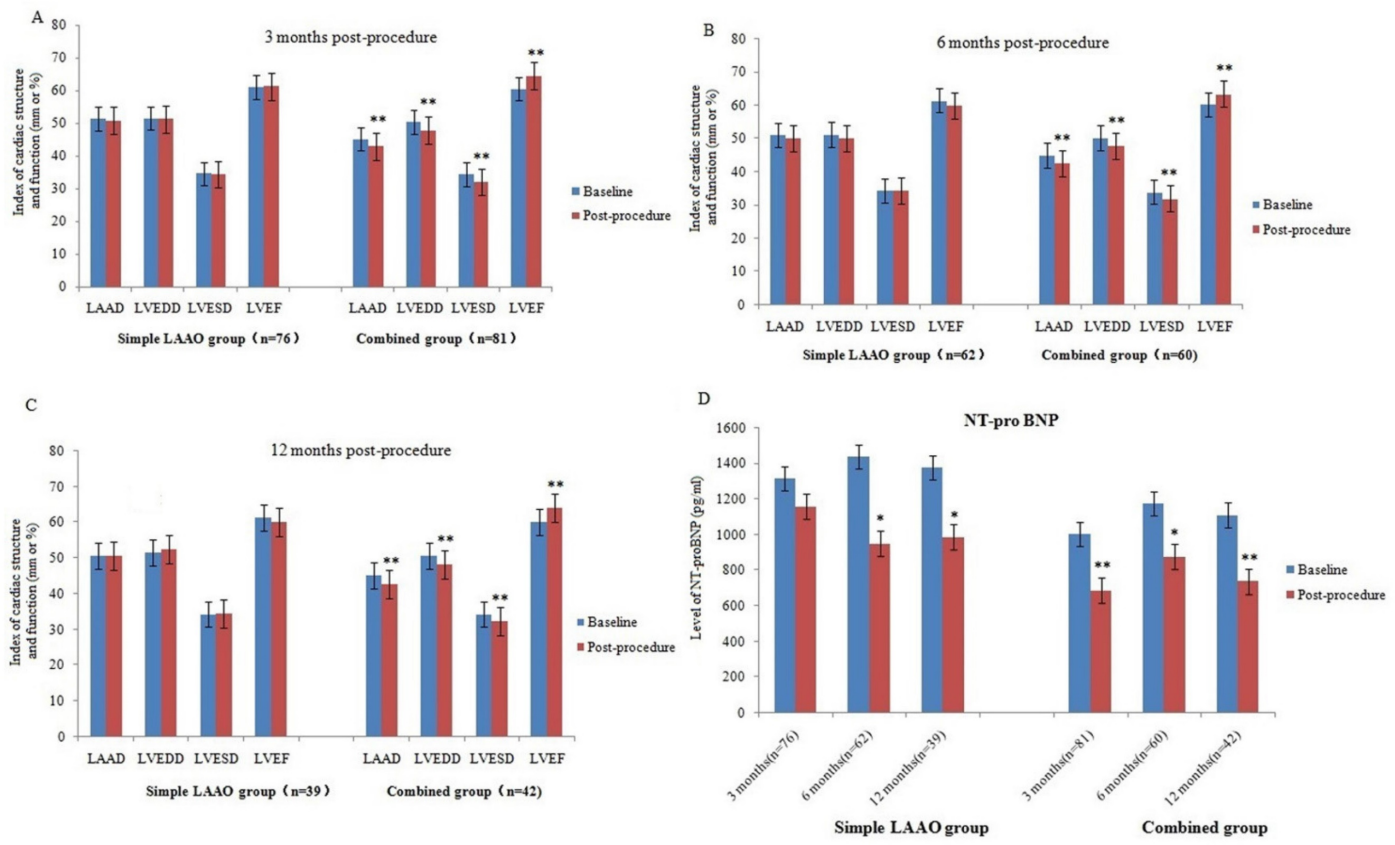
Evolution of cardiac structure and function in simple LAAO group and combined group at 3 months, 6 months and 12 months post- procedure. (A) 3 months post-procedure. (B) 6 months post-procedure. (C) 12 months post-procedure. (D) Change of NT-pro BNP in simple LAAO group and combined group at 3 months, 6 months and 12 months post- procedure. Compared with the baseline, *p<0.05, **p<0.01.

Patients were divided into Simple LAAO group and Combined group according to the procedural approach. Instant PVI with complete LAAO was achieved in combined group. There were 76 patients, 62 patients and 39 patients in simple LAAO group at 3 months, 6 months and 12 months after procedure, while 81 patients, 60 patients and 42 patients in combined group, respectively. Results showed that there was a significant decrease of NT-proBNP levels at 6 months and 12 months after operation in the Simple LAAO group, but not show at 3 months. Meanwhile, no significant change of LAAD, LVEDD, LVESD and LVEF was noted in Simple LAAO group during the 3months, 6 months and 12 months follow-up. However, compared with the baseline, there was significant improvement of LAAD, LVEDD, LVESD, LVEF, NT-proBNP in Combined group at the follow-up periods of 3 months, 6 months, and 12 months. The detailed results are listed in Figure 2.

### Procedure-related complications in follow-up

The Watchman devices of all patients were in place. There were 129 (82.2%) patients, 97 (79.5%) patients and 65 (80.2%) patients without residual flow at 3 months, 6 months and 12 months, while residual flow less than 3 mm were 24 (15.3%) patients, 21(17.2%) patients and 13 (16.1%) patients, respectively. Compared with simple LAAO group, combined group was associated with a significant decrease of no residual flow and increase of residual flow less than 3 mm (p<0.05) at the follow-up of 3 months, 6 months and 12 months. Device-related thrombus was happened in 7 (4.9%) patients, 2 (1.6%) patients and 3 (3.7%) patients at 3 months, 6 months and 12 months after procedure. Correspondingly, 2 (1.3%) cases, 1 (0.8%) case and 2 (2.5%) cases of stroke were noted, respectively. Bleeding complication was observed in 5 cases at 3 months after operation, including 1 case of gastrointestinal hemorrhage, 1 case of hemoptysis, 1 case of urinary system hemorrhage and 2 cases of skin ecchymosis. At 6 months after operation, hemorrhagic complication was occurred in 3 cases, 1 with gastrointestinal hemorrhage and 2 with ecchymosis. At 12 months, gastrointestinal bleeding occurred in 2 cases. 1 case of death occurred at 8 months post- procedure due to sudden cardiac death. Except for the incidence rate of residual flow, there was no significant difference of the procedure-related complications between simple LAAO group and combined group. The results are displayed in Table 4.

## Discussion

At present, the complication of thromboembolism is the leading cause of death and disability in AF, ischemic stroke due to AF accounts for 20% of all strokes 17. It was proven that NVAF-related thrombus was 100% from LAA 18. As a safe and effective therapy of stroke prevention, LAAO provides a viable alternative for oral anticoagulants and has been recommended for the prevention of AF stroke by the guidelines 19.

However, the impact of LAAO on cardiac structure and function in NVAF has been reported seldomly. Meanwhile, there was a controversy on the impact of LAAO on cardiac structure and function in NVAF 9-13. Some studies showed that implantation of a left atrial appendage occluder had no significant effect on cardiac structure and function 9-11. Murtaza G *et al.* found a significant change in left atrial passive emptying fraction and left atrial expansion index after Watchman implantation, which indicated an improvement of the left atrial piping and storage function 12. Phan QT *et al.* found that LAAC led to a remodeling of cardiac functional and structural, which might further facilitate the maintenance of AF and deterioration of heart failure 13. In the present study, there was no significant effect on cardiac structure and left ventricular function over a long-term follow-up after LAAO, except a statistically significant difference for LAAD at 6months post-procedure, which was in line with the previous reports 9, 11. However, the statistically significant difference for LAAD was not noted in simple LAAO group but noted in combined group at 6 months post procedure. Therefore, most of the effect to LAAD at 6 months appeared to result from ablation but not LAAO, which was consistent with J Yang *et al.* 20.

NT-proBNP, a proteolytic precursor with no chemical activity, has a higher sensitivity and half-life than BNP. It was found that there was a significant increase of the ANP and BNP levels immediately after LAAO and decrease at 24 hours post- procedure 21. A study published by Lakkireddy *et al.* showed an immediate increase in NT-proBNP and BNP after LAAO, returning to baseline at 24 h and 3 months 22. In this study, there was a significantly decreased of NT-proBNP levels in total patients and the simple LAAO group at 3 months, 6 month, and 12 months after LAAO, which was consistent with Majunke *et al.* 21. One possible explanation for the decrease of NT-proBNP post procedure may be that the ANP and BNP were increased temporarily by the distraction stimulation of LAA during procedure and decreased due to the decreased secretion of ANP after LAAO. With the gradual endothelialization of the occluder, the secretion of LAA was gradually decreasing and the level of plasma ANP and BNP was decreased further. Another possible reason was that LAAO can reduce LAAD, effectively increase left atrial blood flow velocity, and help improve patient atrial function, which leading to a decrease of plasma ANP and BNP secretion.

As mentioned above, LAAC may have adverse effects on cardiac structure and function. However, RFCA or Cryo may improve cardiac structure and function by restoring and maintaining sinus rhythm. So far, the combination of LAAO for stroke prevention and RFCA or Cryo for AF symptoms control was performed and the safety and feasibility were assessed. The first study on a combined procedure was performed by Swaans *et al.* 23. According to this study, the safety and effectiveness of the combined procedure were confirmed. In addition, the safety and effectiveness of the combined procedure were also confirmed by the first study of the combined Cryo procedure by Fassini *et al.* 24. In our study, instant PVI with complete LAAO was performed in combined group. Meanwhile, there were statistically significant improvements in cardiac structure and function at 3, 6 and 12 months post- procedure in combined group, whereas the procedure -related complications including DRT, stroke and bleeding events were not significantly different from simple LAAO group. Our evidence confirmed the safety and efficacy of the combined procedure and indicated greater benefits could be expected by combined procedure in NVAF patients with clear indications, especially for those NVAF patients with high risk of stroke and heart failure. Of note, in our study, the occurrence of residual flow in combined group was higher than that in simple LAAO group during the follow-up. We hypothesized that a mismatch between LAA and the device due to the edema of pulmonary vein crest after ablation may contribute to this occurrence, which indicated a larger Watchman device should be selected as the suitable size in the consideration of an oversizing by 4 mm-6 mm of the diameter of the LAA.

In conclusion, the main findings in our study are as following: (1) LAAO has no significant effect on cardiac structure and left ventricular function over a long-term follow-up but can significantly reduce the plasma NT-proBNP; (2) LAAO combined with ablation can significantly improve the cardiac structure and function in patients with NVAF. The improvement of cardiac structure and function comes from the influence of ablation, but not LAAO.

### Study limitations

We acknowledge some limitations in the present study. Firstly, the study is a single-center follow-up study with a small sample size and inconsistent selection of patients at each time point, which may be biased and affect the accuracy of the study. Secondly, there are few parameters selected in the study to reflect the cardiac structure and function, and more relevant data are needed to verify the results. Thirdly, the factors such as drugs taken by patients and recurrence of AF may affect cardiac structure and function.

## Figures and Tables

**Table 1:** Baseline characteristics of the population

Baseline characteristics	n=157
Male, n (%)	91 (58.0)
Age (years)	67.4±8.9
BMI (Kg/m²)	25.0±3.4
Paroxysmal AF, n (%)	43 (27.4)
Persistent AF, n (%)	114 (72.6)
CHA2DS2-VASc score	3.06±1.06
HAS-BLED score	2.78±0.81
Medical history	
Hypertension, n (%)	109 (69.4)
Heart failure, n (%)	73 (46.5)
Diabetes mellitus, n (%)	38 (24.2)
Stroke history, n (%)	46 (29.3)
Coronary heart disease, n (%)	35 (22.3)
Abnormal liver function, n (%)	31 (19.7)
Abnormal rennal function, n (%)	4 (2.5)
Hemorrhage history, n (%)	5 (3.2)
Medications	
Antiplatelet agents, n (%)	35 (22.3)
Oral anticoagulants, n (%)	96 (61.1)
Warfarin, n (%)	27 (17.2)
New oral anticoagulants, n (%)	69 (43.9)

Data represented as N (%) or mean ± standard deviation. BMI: body mass index, AF: atrial fibrillation

**Table 2:** The periprocedural parameters

Periprocedural parameters	n=157
Procedure method	
Cryo+LAAO, n (%)	51 (32.5
RFCA+LAAO, n (%)	30 (19.1)
LAAC, n (%)	76 (48.4)
Device size	
21mm, n (%)	11 (7.0)
24mm, n (%)	28 (17.8)
27mm, n (%)	49 (31.2)
30mm, n (%)	30 (19.1)
33mm, n (%)	39 (24.8)
LAA orifice diameter (mm)	
CTA	23.6±3.7**
TEE	22.4±3.6
DSA	22.9±3.7
LAA length (mm)	
CTA	25.1±3.9**
TEE	23.8±3.6
DSA	24.6±3.7
Residual flow	
0mm, n (%)	129 (82.2)
<3mm, n (%)	24 (15.3)
3-5mm, n (%)	4 (2.5)
≥5mm, n (%)	0
Exposure shoulder of occluders	
Yes, n (%)	31 (19.7)
No, n (%)	126 (80.3)
Compression ratio of occlude (%, ‾x±s)	21.6±4.6
Complications	10 (6.4)
Pericardial effusion, n (%)	5 (3.2)
Cardiac tamponade, n (%)	2 (1.3)
Vagal reflex, n (%)	2 (1.3)
Phrenic nerve palsy, n (%)	1 (0.65)

Data represented as N (%) or mean ± standard deviation. Cryo: Cryoballoon ablation, LAAC: Left atrial appendage occlusion, RFCA: Radiofrequency catheter ablation, LAA: Left atrial appendage, CTA: CT angiography, TEE: transesophageal echocardiography, DSA: Digital subtraction angiograph.** Compared with TEE, p<0.01.

**Table 3:** Evolution of cardiac function after procedure

		LAAD (mm)	LVEDD (mm)	LVESD (mm)	LVEF (%)	NT-proBNP (pg/ml)
3 months (n=157)	Baseline	48.22±6.74	50.46±5.84	33.59±6.31	61.75±9.41	1154.30±678.90
	Post-procedure	47.82±6.55	50.51±5.17	33.48±5.64	62.16±9.22	987.30±514.10
	t value	0.533	0.081	0.163	0.391	2.456
	p value	0.125	0.994	0.991	0.338	0.019
6 months (n=122)	Baseline	48.34±6.71	50.63±5.63	33.56±6.32	62.24±9.63	1314.36±698.95
	Post-procedure	46.48±7.07	50.75±5.38	33.16±5.80	61.75±9.06	1127.50±651.90
	t value	2.113	0.171	0.515	0.409	2.159
	p value	0.041	0.533	0.230	0.489	0.036
12 months (n=81)	Baseline	47.75±6.56	50.16±4.82	32.83±5.67	62.96±8.79	1255.00±791.31
	Post-procedure	47.25±6.79	51.30±4.52	33.49±4.83	62.23±9.20	987.00±572.40
	t value	0.476	1.553	0.797	0.516	2.471
	p value	0.107	0.013	0.121	0.444	0.023

Data represented as mean ± standard deviation. LAAD: left atrial anteroposterior diameter, LVEDD: left ventricular end-diastolic dimension, LVESD: left ventricular end-systolic dimension, LVEF: left ventricular ejection fraction, NT-proBNP: N-terminal pro-brain natriuretic peptide

**Table 4:** Follow-up outcomes

	3 months (n=157)	6 months (n=122)	12 months (n=81)
Occluder displace, n (%)	0(0.0)	0(0.0)	0 (0.0)
Residual flow, n (%)			
0mm, n (%)	129 (82.2)	97 (79.5)	65 (80.2)
Simple LAAO group, n (%)	68 (89.4)	54 (87.1)	35 (89.7)
Combined group, n (%)	61 (75.3) *	43 (71.7) *	30 (71.4) *
χ^2^ value	5.37	4.46	4.28
p value	0.01	0.02	0.02
<3mm	24 (15.3)	21 (17.2)	13 (16.1)
Simple LAAO group, n (%)	6 (7.9)	6 (9.7)	3 (7.7)
Combined group, n (%)	18 (22.2)*	15 (25.0)*	10 (23.8)*
χ^2^ value	6.22	5.02	3.90
p value	0.01	0.01	0.03
3-5mm	4 (2.5)	4 (3.3)	3 (3.7)
Simple LAAO group, n (%)	2 (2.6)	2 (3.2)	1 (2.6)
Combined group, n (%)	2 (2.5)	2 (3.3)	2 (4.8)
χ^2^ value	0.00	0.00	0.27
p value	-	-	0.67
≥5mm	0 (0.0)	0 (0.0)	0 (0.0)
Simple LAAO group, n (%)	0 (0.0)	0 (0.0)	0 (0.0)
Combined group, n (%)	0 (0.0)	0 (0.0)	0 (0.0)
χ^2^ value	0.00	0.00	0.00
p value	-	-	-
Device-related thrombus	7 (4.9)	2 (1.6)	3 (3.7)
Simple LAAO group, n (%)	3 (3.9)	1 (1.6)	2 (5.1)
Combined group, n (%)	4 (4.9)	1 (1.7)	1 (2.4)
χ^2^ value	0.09	0.00	0.43
p value	1.27	-	0.49
Stroke	2 (1.3)	1 (0.8)	2 (2.5)
Simple LAAO group, n (%)	1 (1.3)	1 (1.6)	1 (2.6)
Combined group, n (%)	1 (1.2)	0 (0.0)	1 (2.4)
χ^2^ value	0.00	0.98	0.00
p value	-	0.25	-
Bleeding	5 (3.2)	3 (2.6)	2 (2.5)
Simple LAAO group, n (%)	3 (3.9)	1 (1.6)	1 (2.6)
Combined group, n (%)	2 (2.5)	2 (3.3)	1 (2.4)
χ^2^ value	0.28	0.38	0.00
p value	0.65	0.54	-
Death	0 (0.0)	0 (0.0)	1 (1.2)
Simple LAAO group, n (%)	0 (0.0)	0 (0.0)	1 (2.6)
Combined group, n (%)	0 (0.0)	0 (0.0)	0 (0.0)
χ^2^ value	0.00	0.00	0.00
p value	-	-	-
Post procedure medications			
Warfarin, n (%)	24 (15.3)	0 (0.0)	1 (1.2)
Dabigatran, n (%)	106 (67.5)	1 (0.8)	0(0.0)
Rivaroxaban, n (%)	27 (17.2)	1 (0.8)	2 (2.4)
Aspirin, n (%)	126 (80.2)	109 (89.3)	69 (85.2)
Clopidogrel, n (%)	31 (19.8)	122 (100)	12 (14.8)

Data represented as N (%) or mean ± standard deviation. LAAO: left atrial appendage occlusion
